# *Notes from the Field:* Late-Onset Infant Group B Streptococcus Infection Associated with Maternal Consumption of Capsules Containing Dehydrated Placenta — Oregon, 2016

**DOI:** 10.15585/mmwr.mm6625a4

**Published:** 2017-06-30

**Authors:** Genevieve L. Buser, Sayonara Mató, Alexia Y. Zhang, Ben J. Metcalf, Bernard Beall, Ann R. Thomas

**Affiliations:** ^1^Providence Health System, Portland, Oregon; ^2^Randall Children’s Hospital at Legacy Emanuel, Portland, Oregon; ^3^Public Health Division, Oregon Health Authority; ^4^Division of Bacterial Diseases, National Center for Immunization and Respiratory Diseases, CDC.

In September 2016, the Oregon Health Authority was notified of a case of late-onset group B *Streptococcus agalactiae* (GBS) bacteremia in an infant that began 5 days after completion of treatment for early-onset GBS bacteremia. The infant was born at term following an uncomplicated pregnancy; maternal GBS vaginal/rectal screening culture at 37 weeks’ gestation was negative. Shortly after birth, the infant developed signs of respiratory distress and was transferred to the neonatal intensive care unit where blood and cerebrospinal fluid (CSF) were obtained for culture; antibiotics were initiated for presumed sepsis. The blood culture was positive for penicillin-sensitive, clindamycin-intermediate GBS. CSF culture was negative. The infant was discharged and went home after completing an 11-day course of ampicillin (200 mg/kg/day).

Five days later, the infant was taken to the emergency department because of irritability and was admitted to a second hospital. A blood culture yielded penicillin-sensitive, clindamycin-sensitive GBS. CSF was sterile, expressed breast milk did not yield GBS, and serial exams did not reveal a source.

Three days into the infant’s admission to the second hospital, the treating physician was notified by a physician from the birth hospital that the mother had requested release of the placenta at the time of delivery. The mother confirmed that she had registered with Company A to pick up and encapsulate her placenta for ingestion. Three days after the infant’s birth, the mother had received the dehydrated, encapsulated placenta and began ingesting two capsules three times daily. The physician instructed the mother to stop consuming the capsules. A sample of the capsules was cultured, yielding penicillin-sensitive, clindamycin-sensitive GBS. The infant was treated with ampicillin (300 mg/kg/day) for 14 days and gentamicin (3 mg/kg/daily) for the first 6 days and discharged home.

The three GBS isolates (one from each blood infection, and one from the placenta capsules) were indistinguishable by pulsed-field gel electrophoresis. Whole genome sequencing (WGS) performed at CDC revealed no single nucleotide polymorphisms between strains. WGS predicted serotype III, multilocus sequence type 17 (ST17), and tetM+ (tetracycline resistance). The strains had surface-anchored hypervirulent GBS adhesin Hvga, pilus island PI2b, and serine-rich repeat protein Srr2 (*1*); these virulence factors can facilitate adhesion and invasion from the infant’s intestine into the bloodstream and potentially across the blood brain barrier (*2*). Although transmission from other colonized household members could not be ruled out, the final diagnosis was late-onset GBS disease attributable to high maternal colonization secondary to consumption of GBS-infected placental tissue (*3*).

Placenta ingestion has recently been promoted to postpartum women for its physical and psychological benefits, although scientific evidence to support this is lacking (*4*). Placental tissue is consumed raw or prepared by cooking, desiccation, preservation, and other modalities (*5*). Expectant mothers register for Company A’s services before delivery and report preexisting infection with human immunodeficiency virus/acquired immunodeficiency syndrome, hepatitis, herpes, chlamydia, syphilis, and Lyme disease; however, the company does not ask about intra- or postpartum infections. According to Company A’s website, the placenta is cleaned, sliced, and dehydrated at 115°F–160°F (46°C–71°C), then ground and placed into about 115–200 gelatin capsules, and stored at room temperature.

No standards exist for processing placenta for consumption. Heating at 130°F (54°C) for 121 minutes is required to reduce *Salmonella* bacterial counts by 7 log_10_ (*6*). In this case, heating for sufficient time at a temperature adequate to decrease GBS bacterial counts might not have been reached. Consumption of contaminated placenta capsules might have elevated maternal GBS intestinal and skin colonization, facilitating transfer to the infant.

The placenta encapsulation process does not per se eradicate infectious pathogens; thus, placenta capsule ingestion should be avoided. In cases of maternal GBS colonization, chorioamnionitis, or early-onset neonatal GBS infection, ingestion of capsules containing contaminated placenta could heighten maternal colonization, thereby increasing an infant’s risk for late-onset neonatal GBS infection. Clinicians should inquire about a history of placenta ingestion in cases of late-onset GBS infection and educate mothers interested in placenta encapsulation about the potential risks.
